# Secondary Metabolite Gene Regulation in Mycotoxigenic *Fusarium* Species: A Focus on Chromatin

**DOI:** 10.3390/toxins14020096

**Published:** 2022-01-25

**Authors:** Anna Katharina Atanasoff-Kardjalieff, Lena Studt

**Affiliations:** Department of Applied Genetics and Cell Biology, Institute of Microbial Genetics, University of Natural Resources and Life Sciences, Vienna (BOKU), 3430 Tulln an der Donau, Austria; anna.atanasoff-kardjalieff@boku.ac.at

**Keywords:** fungal secondary metabolism, mycotoxins, histone post-translational modifications (histone marks), transcription factors (TFs), gene expression

## Abstract

*Fusarium* is a species-rich group of mycotoxigenic plant pathogens that ranks as one of the most economically important fungal genera in the world. During growth and infection, they are able to produce a vast spectrum of low-molecular-weight compounds, so-called secondary metabolites (SMs). SMs often comprise toxic compounds (i.e., mycotoxins) that contaminate precious food and feed sources and cause adverse health effects in humans and livestock. In this context, understanding the regulation of their biosynthesis is crucial for the development of cropping strategies that aim at minimizing mycotoxin contamination in the field. Nevertheless, currently, only a fraction of SMs have been identified, and even fewer are considered for regular monitoring by regulatory authorities. Limitations to exploit their full chemical potential arise from the fact that the genes involved in their biosynthesis are often silent under standard laboratory conditions and only induced upon specific stimuli mimicking natural conditions in which biosynthesis of the respective SM becomes advantageous for the producer. This implies a complex regulatory network. Several components of these gene networks have been studied in the past, thereby greatly advancing the understanding of SM gene regulation and mycotoxin biosynthesis in general. This review aims at summarizing the latest advances in SM research in these notorious plant pathogens with a focus on chromatin structure.

## 1. Introduction

The genus *Fusarium* belongs to a diverse group of phytopathogenic fungi that collectively can infect virtually all agricultural important crop plants worldwide [1]. Two prominent species, namely *F. graminearum* and *F. oxysporum*, are even ranked amongst the most detrimental plant pathogens worldwide [2]. Some genera are not limited to colonizing plants but may cause severe adverse health effects, i.e., fusariosis, in immunosuppressed patients. These effects are caused by *F. oxysporum* and *F. solani* [3]. In general, the genus *Fusarium* comprises about 300 species found amongst 23 different *Fusarium* species complexes [4,5]. The economically most relevant species belong to four different species complexes: the *F. fujikuroi* species complex (FFSC), *F. graminearum* species complex (FGSC), *F. solani species* (FSSC) complex and the *F. oxysporum* species complex (FOSC) [6,7]. Next to infection, fusaria produce a broad range of low-molecular-weight compounds (so-called secondary metabolites, SMs), including potent toxins that may accumulate during infection [8]. Prominent examples are fumonisins produced by *F. verticillioides*, *F. proliferatum* as well as by some *F. fujikuroi* isolates [9,10,11,12], trichothecenes of class B, e.g., deoxynivalenol (DON) by *F. graminearum* and *F. culmorum* [13], or enniatins and beauvericin produced by numerous *Fusarium* species [14,15]. This not only leads to irreversible effects on plant health and reductions in crop quality but may cause severe intoxication in humans and livestock after the consumption of contaminated food products [16,17]. In light of this matter, it is noteworthy to mention that, to date, only a fraction of fungal SMs are known and subsequently monitored by regulatory authorities.

**Figure 1:**
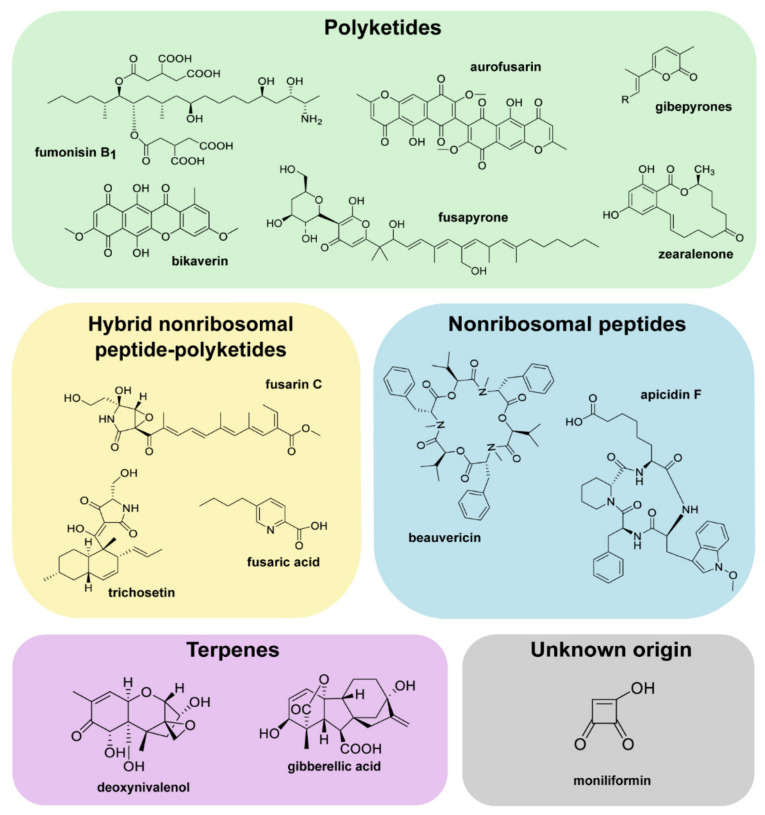
Selection of bioactive *Fusarium* secondary metabolites (SMs). PKS-derived metabolites are shown in the green box, products produced by NRPSs are found in the blue box, and SMs originating from PKS-NRPS hybrids are depicted in the yellow box. TC-derived substances are found in the violet box and moniliformin, with its unknown origin, is shown in the grey box.

In the last decades, vast efforts have been made to obtain more insight into the genetic capacity of various *Fusarium* species via whole-genome sequencing [9]. This has led to the discovery of thousands of unknown genes potentially involved in the formation of novel SMs. Key enzymes involved in this process include polyketide synthases (PKSs), non-ribosomal peptide synthetases (NRPSs), terpene cyclases (TCs), dimethylallytryptophane synthases (DMATSs) or hybrids thereof [9]. In the case of PKSs, fungi usually contain genes belonging to the group of iterative Type I PKSs, which can again be subdivided into highly reducing (HR), partially reducing (PR) and non-reducing (NR) PKSs, depending on their overall domain organization. In very rare cases, fusaria own a set of genes that resembles Type III PKSs [18]. This illustrates the enormous capability of fusaria to produce yet unknown potentially toxic compounds [9,19], and this list is still growing as more genome sequences are published. Examples of prominent bioactive SMs are given in Figure 1.

The biosynthesis of individual SMs is often accompanied by an additional set of enzymes. These are comprised of either tailoring enzymes, which are able to post-translationally modify nascent products such as glycosyltransferases, P450 monooxygenases or oxidoreductases or else transcription factors and/or multidrug transporters involved in regulation and transport, respectively. These genes are often clustered together in the fungal genome, forming SM biosynthetic gene clusters (SMBGCs) and thereby facilitating co-expression under the respective SM-inducing conditions. SMs are mostly dispensable for the survival of the producer but may provide a selective advantage and, as such, may very well become essential for fungal adaption to certain environmental niches [20,21]. This is exemplified by the deletion of *TRI5*, which encodes the deoxynivalenol (DON) biosynthetic key enzyme. This deletion led to attenuated disease symptoms in wheat due to *F. graminearum* [22,23]. Proper disease development is essential for the fungus’ survival through the formation of conidia and ascospores, which serve as the primary inoculum for the next season [24].

The timely expression upon reception of inducing stimuli implies a tight regulatory network. Well-known regulators affecting the biosynthesis of many SMBGCs in *Fusarium* spp. are, for example, the components of the VELVET complex and the associated putative methyltransferase Lae1, which coordinates light, fungal development and secondary metabolism [25,26,27,28,29,30,31,32,33,34,35], or the two GATA transcription factors AreA and Csm1, which connect nitrogen availability and asexual development, respectively, to secondary metabolism [36,37,38,39]. Another level of gene regulation that has emerged as a key player in fungal secondary metabolism over the last decade is based on the chromatin structure, which is thought to enable a swift and reversible expression of a given SMBGC. In this review, we summarize the latest advances in chromatin regulatory mechanisms and their impact on SM gene regulation and mycotoxin biosynthesis in the genus *Fusarium*.

## 2. Chromatin: The Basic Packaging Form of DNA

DNA is present in a highly condensed state in the eukaryotic cell nucleus, mainly to physically package the whole genome, to protect it from damage as well as to control DNA accessibility in a highly regulated manner. The compacted form of DNA is called chromatin, and it consists of about 150 bp of DNA which is tightly wrapped around an octamer of the core histone proteins H2A, H2B, H3, and H4. Chromatin structure differs in their degree of compaction, being either loosely packed and open for transcription (euchromatin) or densely packed and transcriptionally inert (heterochromatin). Heterochromatin can be further distinguished as constitutive heterochromatin (static) and dynamic facultative heterochromatin, which is prone to remodeling processes [40]. As such, it is a natural obstacle for the transcriptional machinery preventing or facilitating gene expression in vivo. Regulatory circuits control chromatin alterations, targeted expression and the silencing of genes under defined (developmental and environmental) conditions. Here, a multifaceted interplay between chromatin-modifying enzymes and transcription factors, or “regulators”, takes place to ensure the metabolites’ timely expression.

The chromatin landscape is subject to a diverse set of modification mechanisms, which results in the alteration of its structure. These regulatory circuits control interactions with the underlying DNA, ensuring the correct biological readout. Already known mechanisms comprise the direct exchange of histones against non-canonical core histones or the removal or addition of whole nucleosome units by energy-dependent chromatin remodeling enzyme complexes [41]. While for the latter, scarce information is available in filamentous fungi thus far, the dynamic exchange of histone variants and the role of H2A.Z has been studied recently in more detail in *F. graminearum* and *F. fujikuroi*. As described for several other organisms, H2A.Z is essential in both fusaria. Intriguingly, lethality is rescued in *F. graminearum* by compensatory mutations in other regulatory subunits of complexes associated with changes in the chromatin landscape, e.g., SWR1, which is important for the dynamic exchange of H2A-H2B dimers against H2A.Z-H2B dimers or the catalytic subunit of Rpd3 complex essential for histone deacetylation [42,43], thereby providing a unique opportunity to analyze the H2A.Z-dependent gene network. Next to this, histone N-terminal tails can experience histone post-translational modifications (histone marks) by the dynamic addition and/or removal of chemical residues, e.g., methyl, acetyl, phosphate or ubiquitin groups. The established signal can then be “read” and relayed by reader proteins, expediting alterations in the chromatin landscape [44,45].

## 3. The Many Shades of Histone Post-Translational Modifications

The first histone mark was already identified by Allfrey and colleagues in 1964 [46], and within subsequent decades, a plethora of novel modifications expanded the known repertoire.

However, so far, only the methylation and acetylation of histone N-termini has been studied in greater detail in filamentous fungi, including *Fusarium*, and will thus be the focus of this review. Whereas histone acetylation is tightly associated with active gene transcription, histone methylation has ambivalent functions. Depending on the exact methylation site and methylation degree, it can either repress or stimulate gene expression [47]. Notably, depending on the *Fusarium* species, the number of predicted histone methyltransferases/demethylases and histone acetyltransferases/deacetylases varies as depicted in Table 1 for selected fusaria of different species complexes. This already implies different regulatory circuits for SM gene expression, even in the same genus. Evidently, this gives rise to the fact that there are most certainly many more histone-modifying enzymes and modifications beyond methylation and acetylation yet to be discovered in the future.

Efforts to alter the chromatin landscape by the manipulation of histone marks to trigger an expressional change of the underlying genes have been manifold across the fungal kingdom. First experiments performed as early as over a decade ago were described for *Aspergillus nidulans*. Here, the deletion of the histone acetyltransferase-encoding gene *hdaA* led to the upregulation of two SM-related genes [49]. Only a little later, the role of histone marks in SM gene regulation was also approached in the genus *Fusarium*. Deletion of the putative H3K9me3 reader protein-encoding gene *FgHEP1* in *F. graminearum* resulted in an altered chemical profile [50]. Since then, many more histone-modifying enzymes, histone marks and associated reader proteins were identified and analyzed in filamentous fungi, including fusaria.

Fusaria are able to produce numerous SMs, and a great effort has been made regarding the identification of novel compounds as well as the linkage of already known SMs to their respective SMBGC. Notably, only a few SMBGCs are conserved among the different *Fusarium* spp. These include, for example, the perithecial pigments (fusarubins) [51], the cyclic hexapeptide fusahexin [52], the siderophore ferricrocin, which is involved in an intracellular iron-capturing metabolite [53], fusarinine, which is important for extracellular iron-chelating [54] and gibepyrones, a group of mycotoxins [55] which are found across the genus. On the contrary, the majority of SMBGCs are only produced by some fusaria. Prominent examples are the natural plant hormone gibberellic acid (GA), primarily produced by *F. fujikuroi* but more seldomly by some *F. proliferatum* or *F. oxysporum* isolates [56,57,58]. The same is true for the mycotoxin DON, which is mainly produced by members of the *F. graminearum* clade and some closely related species [59]. Interestingly, fusaproliferin (FPU) [60] is only known to be produced by members of the FFSC [59]. While the list of identified *Fusarium* metabolites is ever-growing, we are still only at the beginning of understanding the prerequisites of their expression. Table 2 summarizes known *Fusarium* SMs for which biosynthesis and/or gene expression responds to the loss and/or overrepresentation of enzymes involved in altering the chromatin structure in chosen fusaria.

**Figure 2:**
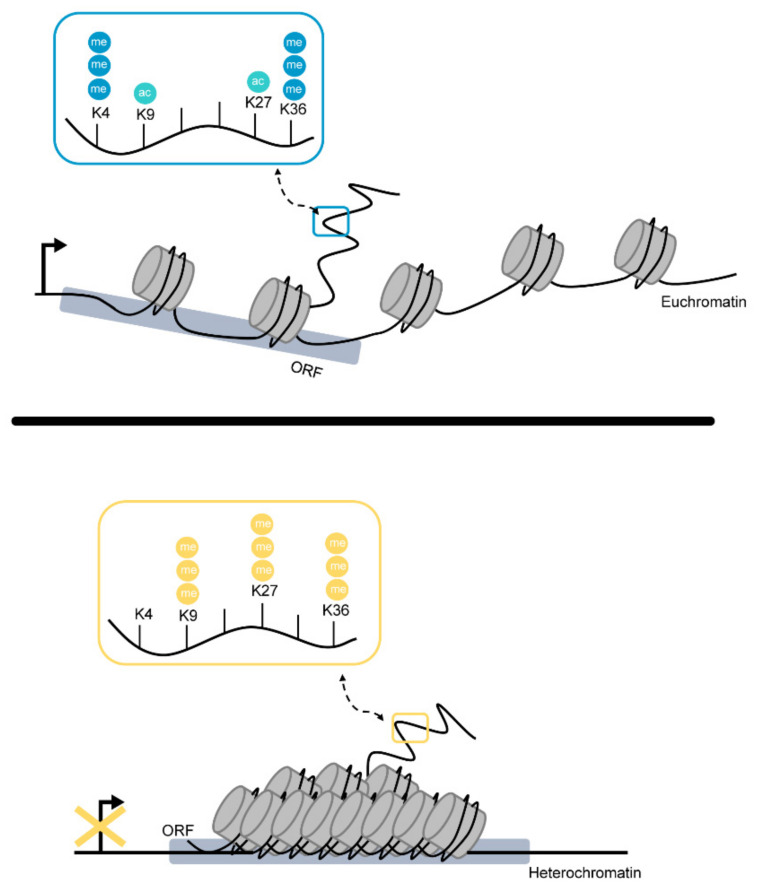
Schematic overview of the chromatin structure and histone marks associated with euchromatin or heterochromatin formation, respectively. In the upper panel, the loosely packed and open for transcription form of chromatin is shown. Here, dark blue beads are recognized as methyl residues, while light blue beads depict acetyl residues on histone N-terminal tails. On the upper panel, the heterochromatic structure is visualized. Yellow beads show methyl residues on prominent histone marks associated with heterochromatin formation.

Histone marks are roughly categorized into groups according to their associated function, i.e., induction of euchromatin formation accompanied by gene expression or gene silencing through compaction and the formation of facultative or constitutive heterochromatin. Thus, some marks are considered heterochromatic, e.g., H3K9me3 or H3K27me3, while others are prone to be associated with euchromatin such as H3K4me3 and histone acetylation on H3/H4. As such, it is noteworthy to mention that, despite the rigid “classical” categorization of histone marks, some own ambivalent characteristics (Figure 2).

The next paragraphs summarize the current knowledge on chromatin regulatory mechanisms, i.e., histone marks, provide an overview on their mechanisms and shed light on the dynamic interplay between so-called global regulators and histone marks with a focus on SM gene regulation in the genus *Fusarium*.

### 3.1. Histone Lysine Methylation—A Ballett of Transcriptional Homeostasis

**Figure 3:**
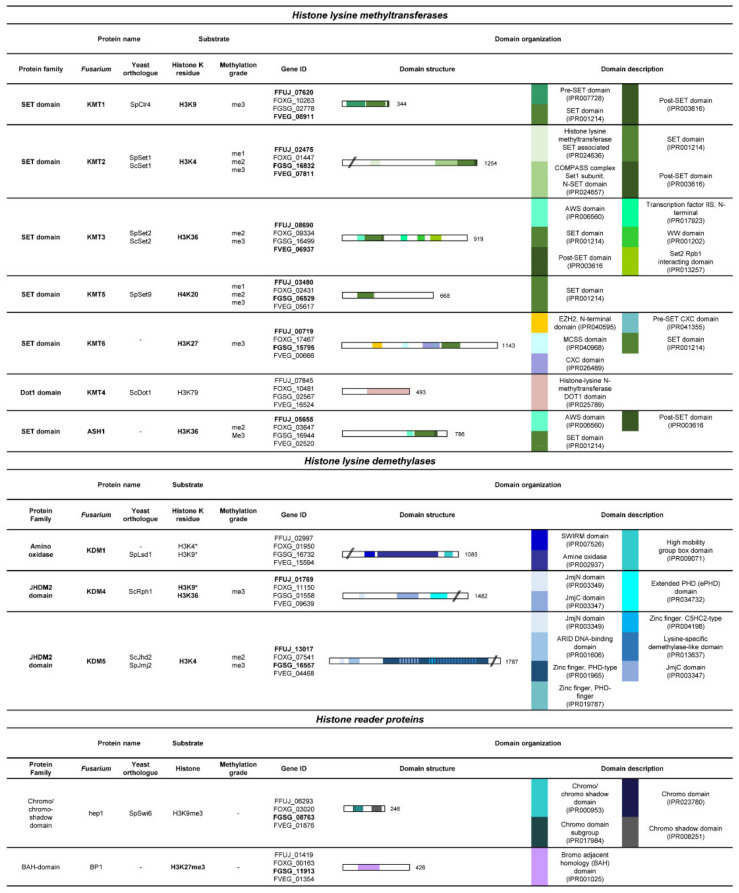
Summary of characterized histone lysine methyltransferases, demethylases and reader proteins in selected fusaria. The table depicts the predicted proteins, their putative functions and their domain organizations. Domain descriptions are based on InterPro [48] and InterPro accession numbers of *F. fujikuroi*, which are given in the table. Accession numbers are written in bold if, in the *Fusarium* species, literature for the respective histone-modifying enzyme is available.

Histone lysine methylation belongs to the most studied histone marks in filamentous fungi, as of now. These marks are generally established by a class of proteins containing a catalytically conserved SET (**S**u(var)3–9, **e**nhancer of zeste and **T**ri-thorax) domain [94], with one exception: DOT1. Thus far, DOT1 is the only described histone lysine methyltransferase without a SET domain [95]. DOT1 structurally resembles arginine methyltransferases but shares conserved motifs with SET domain-containing proteins, enabling DOT1 to solely methylate histone H3 lysine 79 (H3K79me) but not arginine [96]. In contrast, the SET domain recognizes lysine residues at distinct positions on the N-termini, thereby allowing the relocation of one or more methyl groups from S-adenosylmethionin (SAM) to a lysine residue [97]. Depending on the position and degree of methylation, this mark can either lead to transcriptional activation or repression of genes. Thus, the positioning and degree of the installed methylation is critical for the transcriptional read-out [47]. Fusaria harbor around 20–35 putative SET domain-containing proteins, depending on the species (Table 1). Some appear to be conserved between species, whereas others are unique. While the role for most of the SET or DOT domain-containing methyltransferases in the genus *Fusarium* is still enigmatic, some proteins have been studied in greater detail in filamentous fungi, including fusaria. Those are Kmt1 (H3K9me3), Kmt2 (H3K4me3), Kmt3 and Ash1 (H3K36me3), as well as Kmt6 (H3K27me3) (Figure 3).

To safeguard balanced methylation states, methyltransferase’s function can be antagonized by the targeted demethylation of histone lysine residues by another class of enzymes: histone lysine demethylases [98]. The first protein shown to be able to demethylate lysine residues at histone N-termini was the flavin-dependent monoamine oxidase Lsd1 in the fission yeast *Schizosaccharomyces pombe*. SpLsd1 function is limited by the removal of the mono- and dimethylation (me1/me2) states of H3K4/H3K9 residues *via* its amine oxidase domain, at least in *S. pombe* [99]. Despite Lsd1, the largest family of histone demethylases by far is the Jumonji C (JmjC) domain-containing class of enzymes. The recognition of DNA through structural motifs causes a targeted oxidative demethylation reaction that can remove all three states, i.e., mono-, di-, and trimethylation, from its lysine substrate [100,101]. Thus far, only two JmjC domain-containing proteins have been characterized in greater detail in the genus *Fusarium*: Kdm4 (H3K36me) [102] and Kdm5 (H3K4me) [103,104] (Figure 3).

Finally, so-called histone lysine “reader” proteins recognize and process the newly established mark, facilitating the re-structuring of the chromatin structure and assuring the correct readout of the underlying DNA [45]. Bioinformatic analysis predicts a plethora of as-yet mostly uncharacterized “reader” proteins within the genus *Fusarium*. At present, only the putative H3K9me3 and H3K27me3 reader proteins, FgHep1 and FgBp1, respectively, have been characterized in more detail in *F. graminearum* (Figure 3) [50,105].

#### 3.1.1. H3K4me3 Facilitated by Set1/Kmt2 Is a Hallmark of Euchromatin

One of the most intensively studied histone lysine methylation marks so far is the mono-, di- and trimethylation of histone H3 at lysine 4 (H3K4), which is established by components of the so-called COMPASS (**com**plex of **p**roteins **as**sociated with **s**et1) complex [106]. The COMPASS complex consists of 8 subunits in *S. cerevisiae,* i.e., Set1, Bre2, Swd1, Spp1, Swd2, Swd3, Sdc1 and Shg1, with ScSet1 (alternatively Kmt2 [107]) as the catalytic subunit. Overall, the COMPASS complex is responsible for all three methylation states and each subunit is important to establish a distinct global H3K4 methylation pattern [108]. H3K4 methylation is associated with euchromatin formation and, as such, with active gene transcription. However, numerous studies suggest that this mark has ambivalent functions and also localizes to inert heterochromatic regions [106,109]. Homologs of Set1/Kmt2 have been studied in several *Fusarium* spp.

In the rice pathogen *F. fujikuroi* and the cereal pathogen *F. graminearum*, the Set1 orthologs FfSet1 and FgSet1, respectively, are detrimental for H3K4 di- and trimethylation, while remaining levels of H3K4 monomethylation (H3K4me1) were observed for both species [103,110]. SM biosynthesis was de-regulated in strains lacking the catalytic subunit or other COMPASS components, i.e., Bre2/Ccl1 or Sdc1, that are essential for wild-type H3K4me3 levels [110]. This is exemplified by the deletion of *FfSET1*, which revealed an increase in the biosynthesis of the two red pigments bikaverin (BIK) and fusarubins (FSR), as well as fusarin C (FUS) and, to some extent, also fusaric acid (FUB), while production of the plant hormone and virulence factor GA was completely abolished in axenic culture [103]. In line with this, chromatin immunoprecipitation and subsequent quantitative PCR (ChIP-qPCR) in ∆*ffset1* revealed reduced H3K4me2 and me3 levels at the *GA* cluster genes, the only SMBGC in *F. fujikuroi* shown to harbor H3K4me marks under inducing conditions thus far [103]. This phenotype was largely copied in ∆*ffccl1* strains that also resulted in reduced GA production and increased BIK and FUS biosynthesis, while FUB biosynthesis remained unaffected [111]. Notably, while H3K4me2 levels were found to be enriched at both the *GA* but also at the *BIK* genes, levels remained unaffected at the FUS SMBGC [111], suggesting that H3K4me2 alone is not decisive for gene fate [103,111].

Similarly, in *F. graminearum*, the lack of FgSet1 and associated COMPASS components, i.e., FgBre2 or FgSdc1, led to an impaired DON biosynthesis. Again, ChIP-qPCR revealed drastically reduced levels of H3K4me2 and decreased levels of me1- and me3 at the *TRI* cluster genes in ∆*fgset1* [110]. Next to DON, biosynthesis of the red pigment aurofusarin (AUR) was impaired upon loss of FgSet1 accompanied by reduced H3K4me2 levels at *AUR* cluster genes [110]. These findings are largely supported in an independent study on *FgBRE2*, here designated *FgCCL1* [111]. Next to DON, the loss of FgCcl1 led to a significant increase in zearalenone (ZEA) and FUS production, while AUR biosynthesis remained unaffected [111]. Similar to *F. fujikuroi*, H3K4me2/me3 levels are not strictly correlative with the chemical profile. In contrast, global H3K4me2 distribution studies determined by ChIP-seq in *F. graminearum* performed earlier showed that the FUS genes are overall devoid of H3K4me2 in inducing and non-inducing conditions [112]. These results suggest that loss of either Set1 or Ccl1 largely results in the same chemical phenotype of known SMs with few exceptions. It is noteworthy that, despite a drastic decrease of GA and DON biosynthesis in axenic culture, the biosynthesis of both virulence factors appeared remediated during pathogenic growth in ∆*ffccl1* (and to some extent also in ∆*ffset1*) and ∆*fgccl1*, respectively, suggesting that plant signals can still deduce full pathogenicity [111]. Next to *F. fujikuroi* and *F. graminearum*, deletion of *FvSET1* also altered the chemical profile in *F. verticillioides*. Here, biosynthesis of the potent mycotoxin fumonisin B_1_ (FB_1_), the only SM investigated in this study, was significantly impaired upon loss of FvSet1 [113]. To sum up, H3K4 methylation appears detrimental for wild type-like secondary metabolism among the different fusaria, rendering this mark an important regulator for SM biosynthesis. However, while H3K4me2/me3 appear to be present at many euchromatic genes, SMBGCs are mainly devoid of this histone mark [18,112], thereby leaving the exact mode-of-action elusive at this point.

To ensure transcriptional balance, H3K4 methylation can be antagonized by Kdm5 [107], a JmjC domain-containing H3K4me3-specific demethylase, as shown for *F. fujikuroi* and *F. graminearum* [103,104]. Constitutive overexpression of *FfKDM5* showed a decrease of the overall H3K4me3 levels and an increase in global H3K4me2, while deletion of *FfKDM5* led to the opposite [104]. The same is true for FgKdm5 in *F. graminearum*: H3K4me3 was increased, while H3K4me2 was decreased in ∆*fgkdm5*. The amount of the global H3K4me1 was influenced neither by the overexpression nor the deletion of *FfKDM5*. As shown for other organisms, it is most likely that FfKdm1 is involved in the reversal of the me1. This, however, awaits further proof. Next to this, SM analysis supports the antagonizing effects of FfKdm5. Here, deletion of *FfSET1*, as well as constitutive overexpression of *FfKMD5*, showed increased pigment (FSR/BIK) and mycotoxin (FUS) biosynthesis, while ∆*ffkdm5* behaved contrarily.

It is noteworthy that, for both *Fusarium* species, deletion of *KDM5* accompanied by a global increase in H3K4me3 led to the downregulation of most of the SMBGCs investigated here. Though this is in contrast with the proposed activating gene function of H3K4me3, it may very well reflect the fact that the analyzed *Fusarium* SMBGCs are not enriched for H3K4me2/me3 and thus not regulated by H3K4 methylation directly. Another possibility is that Kdm5 has an additional role in regulating SM gene expression apart from its histone demethylase activity as proposed for *A. nidulans* [104,114].

#### 3.1.2. Kmt1 Is Involved in the Establishment of H3K9me3-Dependent Heterochromatin

A prominent mark important for the formation of constitutive heterochromatin—the transcriptionally inert form of chromatin—is histone H3 lysine 9 trimethylation (H3K9me3). H3K9me3 is established by the H3K9-methyltransferase Kmt1 [107], also known as SpClr4 in the fission yeast *S. pombe* [115] or NcDim5 in *Neurospora crassa* [116]. In general, H3K9me3 is mainly located at telomeric and centromeric gene-poor regions and is herewith highly associated with genome maintenance and integrity. In filamentous fungi, H3K9me3 is tightly connected to DNA methylation, which is associated with the deactivation of invasive transposable elements and, as such, with gene silencing, as shown for *N. crassa* [117]. Briefly, DIM-5 is the catalytic subunit of the DCDC (**D**IM-5/-7/-9/**C**UL4/**D**DB1 **c**omplex) complex, which guides DIM-5 to the methylation site and facilitates H3K9me3 [118]. Here, H3K9me3 serves as an anchor recognized by the reader protein heterochromatin protein 1 (HP1) [119], which is thought to act as a ‘barrier’ for euchromatin spread. As a result, HP1 is often found as a component of condensed chromatin [120]. Next to this, in *N. crassa,* HP1 acts as a scaffold for the recruitment of the DNA methyltransferase DIM-2. Here, HP1 can directly interact with DIM-2 through its chromoshadow domain, guiding the methyltransferase to remaining cytosines, which can then be methylated [119,121]. DNA can be subjected to direct methylation on cytosine nucleotides, which is regulated by a family of DNA methyltransferases (DNMTs). Not every organism sports DNA methylation as shown for yeast, and less is known for the genus *Fusarium*.

The function of the DIM-5 homolog Kmt1 has so far only been studied in two fusaria, *F. mangiferae* and *F. verticillioides.* For the latter, deletion of the histone methyltransferase-encoding gene *FvDIM5* showed a strong upregulation of two melanin biosynthesis-related genes on the transcript level and led to an elevated biosynthesis of the mycotoxin FB_1_ [122]. For the mango tree pathogen, *F. mangiferae*, FmKmt1 represses beauvericin (BEA) biosynthesis in nitrogen-limited conditions in basal culture milieu only, while BEA biosynthesis remained unaffected in nitrogen-sufficient and nitrogen-limiting conditions in an acidic milieu. Next to this, a functional FmKmt1 appears essential for wild type-like expression of some PKS-related genes in this fungus. Deletion of *FmKMT1* resulted in downregulation of *FmPKS8* and *FmPKS40*, two (at that time) cryptic PKS genes in *F. mangiferae*. The latter was recently identified as being involved in fusapyrone (FPY) biosynthesis [86]. Notably, genome-wide H3K9me3 distribution as determined by ChIP-seq is only available for *F. fujikuroi* thus far [18]. Here, H3K9me3 appears to be mainly located at pericentric and centromeric regions, and none of the 48 putative SMBGCs fall within H3K9me3-enriched regions [18]. Thus, it remains opaque at the moment how H3K9me3 mediates SM gene regulation in the genus *Fusarium*.

Other components involved in constitutive heterochromatin formation have been characterized in *F. graminearum*. Here, loss of the HP1 ortholog FgHep1 had no impact on global H3K9me3 levels, but SM analysis showed decreased DON biosynthesis, while AUR biosynthesis was significantly increased in axenic cultures [50]. Recently, the two DNA methyltransferase-encoding genes, *FgDIM-2* and *FgRID*, were identified and characterized in *F. graminearum* [123]. Chemical analysis of single deletion mutants, ∆*fgdim-2* and ∆*fgRID*, revealed elevated levels of 15-acetylated DON (15-ADON) for both, and a strain deleted for both, ∆∆*fgdim-2*/*fgRID*, led to an even more pronounced biosynthesis of this mycotoxin. Next to this, transcriptomic analysis of the double deletion strains suggests that additional SM-associated genes are also affected by the loss of FgDim-2 and FgRID. Concomitantly, SM measurement showed a high correlation between metabolic and transcriptomic data. In detail, the ∆∆*fgdim-2/fgRID* double deletion mutant was enriched in DON, fusaoctaxin and gramilin biosynthesis, while a decrease in AUR, FUS and fusaristatin production was observed [123]. Notably, deletion of both DNTMs (∆∆*fgdim-2*/*fgRID*) did not completely abolish DNA methylation in this fungus, suggesting the presence of more DNA methyltransferases in *F. graminearum*. 

To sum up, H3K9me3 is involved in SM gene regulation in the genus *Fusarium* in some way. However, future research on the different components involved in the formation of constitutive heterochromatin in one organism combined with genome-wide H3K9me3 distribution analysis is required to shed some light on this issue [123].

#### 3.1.3. H3K27me3 Is a *Bona Fide* Mark for Facultative Heterochromatin Installed by Kmt6

The tri-methylation of histone H3 lysine 27 (H3K27me3) is a mark of repressive chromatin domains and is typically associated with chromosomal regions subject to a dynamic regulation, also called facultative heterochromatin [124]. This mark is established by the lysine methyltransferase Kmt6 [107], the homolog of **E**nhancer of **z**este (E(Z)) in *Drosophila melanogaster* [125] and Set7 in *N. crassa* [126]. Kmt6 is a part of the **p**olycomb **r**epressive **c**omplex-2 (PRC2), which in *D. melanogaster* consists of four core proteins: E(Z), **su**ppressor of **z**este **12** (SU(Z)12), **e**xtra **s**ex **c**ombs (ESC) and Nurf55. All components of the PRC2 complex are present in the genus *Fusarium*; however, the Nurf55 ortholog most likely acts outside the PRC2 complex, at least in *N. crassa* and *F. graminearum* [105,126]. Next to Kmt6, Suz12 and Eed (ESC) are critical for H3K27 methylation in *F. graminearum* [105], which agrees with previous studies in *D. melanogaster* [127] or *N. crassa* [126].

In contrast to *N. crassa* [126], large genomic regions are decorated with H3K27me3 in the plant pathogens *F. fujikuroi* and *F. graminearum* [112,128]. Intriguingly, many of the SMBGCs reside in these H3K27me3-rich regions, suggesting that H3K27me3 is an important regulatory layer in controlling fungal SM gene expression. Indeed, the deletion of *FgKMT6* as well as the knock-down of *FfKMT6* in *F. graminearum* and *F. fujikuroi*, respectively, resulted in the de-repression of 15–30% of all genes, including many formerly silent and cryptic SMBGCs in both species [112,128]. In the case of *F. graminearum*, 35 out of 45 SMBGCs are enriched in H3K27me3 in axenic culture. Lack of FgKmt6 led to the de-repression of 32 of these 35 SMBGCs. Similarly, for *F. fujikuroi,* 20 out of 48 putative SMBGCs were affected by the partial relief of H3K27me3 [128]. In line with this, induction of the (formerly) cryptic DMATS3-, PKS-NRPS1-, NRPS22- and STC5-encoding genes was accompanied by reduced H3K27me3 and the accumulation of novel (formerly) cryptic compounds in the *FfKMT6* knock-down strains [91,128]. Similar to *F. graminearum*, a mass spectrometry (MS)-based metabolomics approach identified overall 22 SMs in the *fgkmt6* deletion mutant that were absent from cultures of the corresponding wild-type strain [129]. To sum up, H3K27me3 mediated by Kmt6 is an important regulatory layer for SM gene-silencing in *Fusarium*.

PRC1 components are described to “read” H3K27me3 deposited by PRC2 and, by this mechanism, promote chromatin compaction and maintenance of heterochromatic structures, as shown for *D. melanogaster* [130]. However, PRC1 components are absent from filamentous fungi, thereby implying a different route [131]. Only recently, the BAH domain-containing PHD-protein 1 (FgBP1) was identified that was shown to bind H3K27me3 in vivo. FgBp1 localizes to the nucleus and is able to simultaneously bind H3K27me3 peptides and interact with the chromatin structure *via* its PHD domain, thereby facilitating distinct changes to the underlying chromatin landscape. RNA-sequencing (RNA-seq) analysis determined that loss of FgBp1 leads to the de-repression of a gene set, which is about 94% similar to the *FgKMT6* deletion mutant. Comparable to ∆*fgkmt6*, about 74% of all genes associated with fungal secondary metabolism were strongly up-regulated, which goes in line with FgBP1 being involved in reading H3K27me3. Unexpectedly, only 44% of the genes bound by FgBP1 were detected in regions enriched for H3K27me3, entailing putative secondary functions of this protein [105]. This, as well as its function in other fungi harboring a PRC2 complex, awaits further proof in the future.

#### 3.1.4. Two Enzymes, Kmt3 and Ash1, Are Involved in H3K36me3

As some histone marks can strictly be associated with euchromatic or heterochromatic regions, it is more difficult for histone H3 lysine 36 trimethylation (H3K36me3). H3K36me3 is known to be located in both transcriptional regions in filamentous fungi [102]. H3K36 mono-, di- and trimethylation is established by a single methyltransferase ScSet2 [132] or Kmt3 [107] in *Saccharomyces cerevisiae*. Here, it is shown that the hyperphosphorylated form of RNA polymerase II (RNA PolII) travels along with ScSet2 and acts as a potent activator but also repressor of gene transcription [133,134].

For the genus *Fusarium*, the first studies were performed in the maize pathogen *F. verticillioides*. Here, the absence of FvSet2 led to reduced amounts of the mycotoxin FB_1_ and the red pigment BIK in axenic culture, accompanied by reduced levels of H3K36me3 at *FUM* and *BIK* cluster genes as determined by ChIP-qPCR [135]. Unexpectedly, deletion of *FvSET2* in *F. verticillioides* led to a drastic reduction but not the complete abolishment of H3K36me3 levels in Western blot analysis [135]. These residual levels suggest that another histone methyltransferase is also involved in the methylation of H3K36. Only a little later, this assumption was verified in *F. fujikuroi*, where a second SET-domain containing protein, FfAsh1, was identified to be also involved in methylating H3K36 next to FfSet2 [102]. While H3K36me3 was still detectable in either ∆*ffset2* or ∆*ffash1*, H3K36me3 was completely abolished in ∆∆*ffset2*/*ffash1* in this species. While H3K36me3 is found along complete chromosome arms [102,112], both enzymes methylate discrete chromosome regions: FfSet2-mediated H3K36me3 contributes to regions associated with euchromatin, whereas FfAsh1-mediated H3K36me3 can be found mainly in sub-telomeric regions. [102,112]. Microarray analysis of both single mutants revealed that most (known as well as unknown) SM key enzyme-encoding genes depend on FfSet2 and/or FfAsh1. It is noteworthy that BIK biosynthesis was distinctly affected in *F. fujikuroi* compared to *F. verticillioides*: while in the latter, FvSet2 is involved in BIK biosynthesis, deletion of *FfASH1* but not *FfSET2* affects BIK production [102]. Chemical analysis determined impaired GA, FUS and FUB production in both deletion strains, while BIK biosynthesis was only affected by the loss of FfAsh1 but not FfSet2. Decreased levels of BIK and GA were accompanied by an increase in H3K27me3 at the respective cluster genes as determined by ChIP-qPCR only in the *FfASH1* deletion mutant. Intriguingly, both clusters are located at subtelomeric regions, generally targeted by FfKmt6 and FfAsh1, but Set2 appears to mediate H3K36me3 at the BIK cluster [128]. However, no correlation between H3K36me3 and H3K27me3 was found regarding fungal secondary metabolism in general in *F. fujikuroi* [102].

H3K36me3 is removed by ScRph1 or Kdm4 [102,136], a member of the JmjC domain-containing protein family in *S. cerevisiae* [136,137]. Deletion of *FfKDM4* in *F. fujikuroi* did not reveal an overall increase in H3K36 methylation and did not affect the biosynthesis of known SMs, whereas the constitutive overexpression of *KDM4* showed reduced genome-wide H3K36me3 as determined by Western blot, thereby confirming FfKdm4 as a H3K36-specific demethylase. Of note, FfKdm4 overexpression is unstable, thereby prohibiting further phenotypic characterizations [102]. Whether this phenomenon is related to H3K36me3 or another histone mark, e.g., H3K9me3 or H3K27me3, remains to be elucidated, but it is noteworthy that both histone marks appear to be essential in *F. fujikuroi* [128].

#### 3.1.5. Kmt5-Mediated H4K20me3—A Histone Mark to Be Explored

Histone H4 lysine 20 trimethylation (H4K20me3) is a repressive histone mark and, as such, is associated with heterochromatin formation and herein gene silencing in higher eukaryotes [138]. Other in-depth studies in the fission yeast *S. pombe* implicated that H4K20me is more restricted to coordinating the DNA damage response than heterochromatin formation [139,140]. All three states of H4K20 methylation (me1/me2/me3) are established by SET9 or Kmt5 [107] in *S. pombe* [140].

Thus far, H4K20 methylation is not well understood in filamentous fungi, and only recently was the role of Kmt5 in secondary metabolism explored in the two plant pathogens *F. graminearum* and *F. fujikuroi* [11]. As observed for *S. pombe*, in fusaria, Kmt5 is solely responsible for the me1, me2 and me3 of H4K20. Deletion of *FgKMT5* and *FfKMT5* has distinct effects on fungal secondary metabolism, depending on the species. Deletion of *FfKMT5* positively affects FUS biosynthesis in *F. fujikuroi*, while the same pathway was negatively affected by ∆*fgkmt5* in *F. graminearum*. While the loss of FgKmt5 led to strongly reduced amounts of ZEA, constitutive overexpression of *FgKMT5* led to a slight but significant upregulation of DON biosynthesis. In *F. fujikuroi*, *FfKMT5* overexpression positively influenced GA and BIK biosynthesis [141]. Notably, genome-wide distribution of H4K20me3 is currently unknown in filamentous fungi, including *Fusarium*, and hence their role in regulating fungal SMBGCs remains elusive at the moment.

### 3.2. Histone Lysine Acetylation

Histone methylation generally is recognized by reader proteins, determining the putative readout of the newly established mark, i.e., formation of condensed or open chromatin, while histone acetylation itself changes the chromatin formation through neutralization of the positively charged lysine residues, resulting in the overall relaxation of the chromatin structure [142]. The acetylation process is mediated by various enzyme complexes containing a histone acetyltransferase (HAT/KAT) as the catalytic subunit [40]. Briefly, the addition of an acetyl group leads to the relaxation of the chromatin structure and serves as a molecular tag for the recruitment of chromatin-modifying complexes, thereby facilitating the transcription of prior silenced genes [143,144]. For example, in *F. fujikuroi*, H3K9ac is highly abundant at transcribed genes, including active SMBGCs, while this histone mark is largely absent from non-expressed genes [18]. HATs are divided into nuclear A-type or cytoplasmic B-type acetyltransferases, depending on their localization and targets. Nuclear A-type HATs are further categorized into different subfamilies, i.e., GNAT (**g**eneral **c**ontrol **n**on-de repressible 5 (GCN5)-related **a**cetyl**t**ransferase), MYST (**M**OZ, **Y**bf2/Sas3, **S**as2, and **T**IP60) and Rtt109 (**r**egulator of **T**y1 **t**ransposition gene product **109**) [145], with the superfamily of GNAT being by far the largest with around 60 to almost 100 predicted members in fusaria (Table 1). Thus far, only the GNAT member Gcn5 has been characterized in greater detail in filamentous fungi, including *Fusarium* [146,147,148], and even less information is available on members of the MYST or Rtt109 family. Notably, most often, gene expression is controlled by governed subunits of multimeric complexes, such as the SAGA or Nua4/3 complexes. Consecutive dissection of those complexes was not performed in detail so far in the genus *Fusarium* but included in the following paragraphs, emphasizing their importance regarding SM gene regulation.

**Figure 4:**
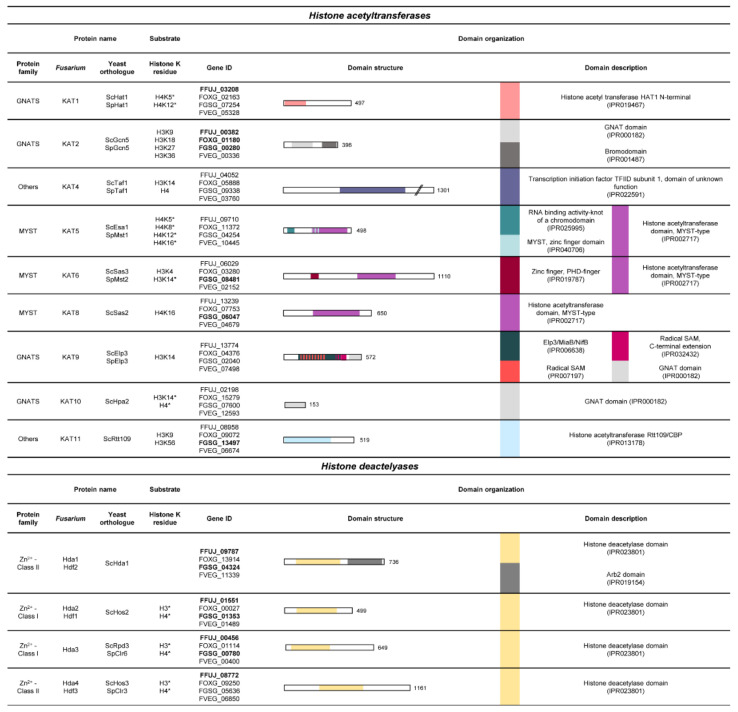
Summary of characterized histone lysine acetyltransferases in selected fusaria. The figure shows the predicted protein, their putative functions and their domain organizations based on *F. fujikuroi*. Domain descriptions are based on InterPro [48], and InterPro accession numbers are given in the table. Accession numbers are written in bold if, in the *Fusarium* species, literature for the respective histone-modifying enzyme is available. * donates to data collected from experiments performed in yeast.

While hyperacetylation of histone proteins is strongly associated with gene transcription, the removal thereof is associated with gene repression [149]. Acetyl groups are actively removed by histone deacetylases (HDACs), resulting in hypoacetylation and hence a more condensed chromatin state [150]. In general, HDACs are grouped into two superfamilies depending on their mode of action: Class I and II Zn^2+^-dependent HDACs as well as Class III NAD^+^-dependent **s**ilent **i**nformation **r**egulators **2** (Sir2) (“sirtuins”) [150]. For the latter, only scarce information is available in filamentous fungi. Therefore, their function remains so far mainly enigmatic. In contrast, Zn^2+^-dependent HDACs have been characterized in more detail in fusaria (Figure 4). So far, the number of HDACs present in different fusaria is limited (Table 1), which inevitably raises the question of whether those exhibit targeted or substrate-specific deacetylation.

#### 3.2.1. The SAGA Complex

The SAGA (**S**pt-**A**da-**Gc**n5-**a**cetyltransferase) complex is one of the most studied chromatin-modifying complexes in filamentous fungi, including fusaria. In *S. cerevisiae,* the SAGA complex consists of 18 highly conserved subunits, including a structural core, the catalytic acetyltransferase Gcn5 (KAT2) [107], a histone de-ubiquitinase (DUB) and an activator-binding module [151,152].

For *F. fujikuroi*, co-immunoprecipitation (co-IP) determined the presence of all structural elements known from yeast, except the DUB submodule Chd1 as well as the physical interaction of FfGcn5 and the transcriptional adaptors FfAda2 and FfAda3. Demonstrating the manifold substrate recognition, deletion of *FfGCN5*, *FfADA2,* and *FfADA3* revealed that they are detrimental at least for H3K4, H3K9, H3K18, H3K27 and H3K36 acetylation [146]. In *F. graminearum* a similar but still distinct Gcn5-dependent acetylation pattern was observed. Here, loss of FgGcn5 resulted in the dramatic reduction of H3K9, H3K14, H3K18 and H3K27 acetylation [148]. Overall, deletion of *GCN5* led to the de-regulation of a large set of genes, including primary and secondary metabolism in *F. fujikuroi*, *F. graminearum* and *F. oxysporum* [147]. In detail, for *F. graminearum*, 202 genes belonging to 62 SMBGCs were affected in the ∆*fggcn5* mutant, including *TRI* gene expression. Concomitantly, DON biosynthesis was almost abolished upon deletion of *FgGCN5* [148]. Four conserved motifs in the N-terminus, as well as the bromodomain on the C-terminus of FgGcn5, were found to be critical for DON biosynthesis [148,153]. In line with these results, the application of the chemical compound phenazine-1-carboxamide (PCN), which is able to target and inhibit FgGcn5 function in vivo, resulted in impaired DON production [154]. Notably, using PCN as a biocontrol to inhibit *Fusarium*-related disease on crop plants is tempting, but since the SAGA complex is conserved from yeast to humans, the effect of PCN on non-target organisms must be evaluated in detail. For *F. fujikuroi*, *FfGCN5* deletion resulted in the deregulation of 61% of all SM key enzyme-encoding genes. Here, the majority of these genes were down-regulated in at least one condition, going in line with the activating role of histone acetylation [146]. To study histone acetylation in more detail, ChIP-qPCR analyses were performed under nitrogen sufficient conditions (60mM glutamine) of selected SMBGCs (GA/BIK/FUB) in the ∆*ffgcn5* mutant. As expected, H3K9, H3K14, and H3K27 acetylation levels were barely detectable at the 5′-end of those SMBGCs. Interestingly, transcriptomic data suggests decreased GA and BIK biosynthesis under these normally repressing conditions, while deletion of *FfGCN5* had no impact on FUB biosynthesis. For GA and BIK, it is doubtful that under non-inducing conditions, histone acetylation is present anyways, while for FUB biosynthesis, loss of these acetylation marks had no impact on biosynthesis, suggesting the involvement of another regulator. Genome-wide ChIP analyses of each acetylation mark under inducing/non-inducing conditions would be more conclusive at this point [146]. To sum up, the SAGA complex is conserved from yeast to humans and assumes important functions regarding global histone acetylation levels. It is essential for the acetylation of several lysine residues in *Fusarium*, and additional target sites are likely. Not surprisingly, SM gene regulation is affected by the loss of Gcn5. However, further studies are required to dissect the impact of single acetylation marks on SM gene expression and thus unravel how Gcn5 and the SAGA complex mediate SM gene expression in this fungus.

#### 3.2.2. The NuA4 and NuA3 Complex

The NuA4 complex consists of 13 unique subunits important for nucleosome acetylation, transcription and recruitment of other factors [155]. Esa1 belongs to the MYST superfamily of histone acetyltransferases and is the only histone acetyltransferase known so far that is essential in the yeast *S. cerevisiae* [156]. This is also true for higher eukaryotes, e.g., *A. nidulans* or *F. graminearum* [157,158]. In *S. cerevisiae,* ScEsa1 is the catalytic subunit of the NuA4 (**Nu**cleosomal **A**cetyltransferase of histone H**4**) complex, important for global acetylation levels on H4 [159]. Thus far, its role in filamentous fungi and secondary metabolism is largely enigmatic.

One conserved component of the NuA4 complex is ScYng2, which is responsible for transcriptional activation in yeast. Contrary to FgEsa1, the ScYng2 ortholog, Fng1, is dispensable in *F. graminearum* [160]. Deletion of *FgFNG1* revealed that it is integral for the function and stability of the NuA4 complex, and as such, FgFng1 is detrimental for the acetylation of H4K5, H4K8 and H4K12 and to a lesser extent also for H4K16. Localization studies showed that FgFng1 is enriched in euchromatic regions co-localizing with H3K4me2. Next to this, transcriptomic data of a ∆*fgfng1* deletion strain showed a strong upregulation of FgKmt2 (FgSet1) and FgKmt6. However, no direct correlation between H4 acetylation and H3K4- or H3K27 methylation has been confirmed so far. Notably, the *FgFNG1* deletion strain is defective in DON biosynthesis [158].

Sas3 (**s**omething **a**bout **s**ilencing protein), also known as KAT6 [107], belongs to the MYST superfamily of acetyltransferases and is the catalytic subunit of NuA3 (**nu**cleosome **a**cetyltransferase of histone H**3**). It is important for gene transcription, HAT activity and integrity of the complex in *S. cerevisiae*. [161,162]. In *F. graminearum,* FgSas3 is important for the acetylation on H3K4 and H3K14, but the loss of FgSas3 implicated no change on global H3 acetylation levels. Deletion of *FgSAS3* revealed the deregulation of important SM genes involved in AUR, ZEA and DON biosynthesis [148].

The accessory factor Eaf6 is a component of the NuA4/NuA3 complex. Characterization of FgEaf6 in *F. graminearum* revealed reduced virulence on wheat plants, though in a DON-independent manner [163], suggesting that other important pathogenicity-inducing genes are impacted by the loss of FgEaf6.

#### 3.2.3. The Roles of So Far Less Characterized Histone Acetyltransferases

The roles of other histone acetyltransferases, Sas2, Elp3 and Rtt109, in fungal secondary metabolism is mainly opaque in the genus *Fusarium*. Only in *F. graminearum* little information on their function and role in secondary metabolism is available as of now.

Sas2 (**s**omething **a**bout **s**ilencing **2**), or KAT8, [107] is the catalytic subunit of the SAS/HAT complex and, as such, is a member of the MYST superfamily. Here, Sas2 plays a critical role in telomeric silencing and H4K16 acetylation in *S. cerevisiae* [159,164]. Sas2 is not important for DON biosynthesis in *F. graminearum*, and its deletion also had no significant impact on the biosynthesis of other known SMs [148].

Elp3 (or KAT9 [107]) is the catalytic component of the Elongator complex and belongs to the GNAT family of acetyltransferases [107]. Its function is associated with the acetylation of H3K14 and H4K8/K12 [165,166]. Deletion of *FgELP3* resulted in slightly disturbed H3K14 acetylation levels and was accompanied by abolished DON biosynthesis in *F. graminearum* [167]. It is tempting to speculate that since the deletion of *FgGCN5* and *FgSAS3* led to a strong reduction but not complete loss of H3K14 acetylation, FgElp3 partially contributes to global H3K14 levels by an unknown mode of action in *F. graminearum*. Notably, deletion of *FfGCN5* did not alter H3K14 acetylation levels in *F. fujikuroi* [146,148], suggesting distinct targets for the HATs in the different fusaria.

Differently to Sas2 and Elp3, Rtt109 (**r**egulator of **T**y1 **t**ransposition **109**)/KAT12 [107] is a fungal-specific histone acetyltransferase responsible for acetylation on histone H3 at K9, K27 and K56 in yeast [168]. Recent studies in *Aspergillus flavus* and *Beauveria bassiana* imply acetylation activity at H3K9 and/or H3K56 residues, respectively [169,170]. Loss of FgRtt109 had no significant impact on DON biosynthesis in *F. graminearum* [148]. It remains to be determined whether Sas2, Elp3 and Rtt109 have similar functions in the other *Fusarium* spp.

#### 3.2.4. HAT1—A Standalone Histone Acetyltransferase

The yeast ortholog ScHat1 belongs to the class B of histone acetyltransferases and is shown to solely post-translationally modify lysine 5 and 12 of the free histone H4 [171,172]. Next to this, Hat1 is not a part of a multimodule enzyme complex, but rather is associated with another single factor, Hat2, that is important to stabilize and enhance Hat1 function [172,173].

For *Fusarium* species, the Hat1 (KAT1) [107] function is currently mainly opaque; only a little information is available for FfHat1 in *F. fujikuroi*. Here, localization studies revealed, that FfHat1 is mainly localized to the nucleus, unlike what has been proposed for class B HATs. This, however, is in agreement with reports from *S. cerevisiae* [34,171]. FfHat1 positively affects GA biosynthesis in *F. fujikuroi*, as targeted deletion and constitutive overexpression of *FfHAT1* showed reduced and elevated GA levels, respectively. In contrast, FUB biosynthesis appears to be negatively affected by FfHat1, as FUB levels were elevated in the *FfHAT1* deletion strain, while the opposite was observed in the overexpression strain [34]. It remains to be investigated which histone marks are associated with Hat1 function in *Fusarium*.

#### 3.2.5. The Group of Zn^2+^-Dependent HDACs: Class I and II

Histone deacetylases counteract histone acetyltransferase function by the dynamic removal of acetyl residues, not only altering chromatin structure but leading to the addition or exclusion of alternative histone marks. Zn^2+^-dependent HDACs of Class I and II are well characterized in yeast and humans and sport many important functions regarding gene expression [142]. This class of HDAC has been characterized in more detail in *F. fujikuroi* and *F. graminearum* and, to some extent, also in *F. asiaticum*. Hda1, Hda2 and Hda4 are dispensable, while the homolog of Rpd3, Hda3, appears essential in both species [174,175]. A HDAC activity assay showed that FfHda1 and FfHda2 contribute to 64% and 25% of overall HDAC activity in *F. fujikuroi*, respectively. Microarray analysis for *ffhda1* deletion mutants showed a strong down-regulation of all investigated genes, including 10 out of 48 SMBGCs. Subsequent SM analyses confirmed microarray analysis for selected SMs. Similar results were observed for the ∆*ffhda2* mutant, while deletion of *FfHDA4* did not reveal changes in its SM profile. Interestingly, constitutive overexpression of *FfHDA1* showed a similar chemical profile as the respective deletion strain, suggesting that balanced acetylation levels are crucial for wild type-like secondary metabolism. Notably, in *F. graminearum,* deletion of *FgHDF1* (Hda2 ortholog) led to an overall reduction of 60% of HDAC activity, possibly implying different roles for the HDACs in both species [174,175]. In *F. graminearum,* lack of FgHdf1 positively influenced AUR biosynthesis, while slightly decreased DON levels were detected [174]. Interestingly, loss of FaHdf2 (Hda1 ortholog) in *F. asiaticum* resulted in significantly increased DON production, while no impact on DON production was observed for the *FgHDF1* deletion strain [174,176].

Next to already known compounds, deletion of *FfHDA1* induced expression of the otherwise silent *NRPS22* gene cluster, which was later identified to be responsible for the biosynthesis of the toxic depsipeptide BEA [9,69]. Detailed analysis of the *BEA* gene cluster revealed that loss of FfHda1 was accompanied by increased H3K27 acetylation at BEA cluster genes [69]. This suggests that FfHda1, in addition to FfKmt6, is crucial for silencing *BEA* gene expression under standard laboratory conditions [69,128,175]. While at least some targets are known for the histone acetyltransferases, less is known for HDACs, but they are generally not as substrate-specific as KMTs. In *F. fujikuroi,* H3K9ac, present under inducing conditions in the wild type, was found to be reduced at the *GA* gene cluster in ∆*ffhda*1, in line with a decreased biosynthesis under inducing conditions. Similarly, H3K9ac was enriched at *BIK* genes under non-favorable conditions accompanied by BIK production upon loss of FfHda1. On the contrary, decreased BIK production was observed under favorable conditions, suggesting that additional factors are involved and necessary for the correct biological read-out. HAT complexes are known to recruit global transcription factors (TFs); the disturbance of the acetylation level could partly overrule this phenomenon [175].

Thus far, the functions of Zn^2+^-dependent HDACs are largely enigmatic, and their overall targets are unknown. In the case of sirtuin-domain HDACs, no information is available at all in the genus *Fusarium*, giving the incentive to analyze both classes of histone-modifying enzymes and their interplay with other histone marks in the future to shed light on their mode-of-action in vivo.

## 4. The Dynamic Interplay of Histone-Modifying Enzymes, Global and Narrow Regulators

Changes in the chromatin structure are mediated through altering the composition of the histone marks, thereby giving way to other factors to act on the underlying DNA, such as narrow- or broad-domain TFs. This illustrates a complex gene network involving the establishment and removal of histone marks, the recruitment of TFs and, finally, accessibility for PolII, thereby ensuring the appropriate changes in the chromatin structure and the correct biological readout of the underlying DNA. Few examples exist that shed light on this complex topic.

In general, filamentous fungi can utilize a diverse set of nitrogen sources but use energetically favored substrates, e.g., ammonium or glutamine, before scavenging alternative substances [177]. Two GATA -type TFs, namely AreA and AreB are involved in this regulatory circuit. AreA is crucial for the de-repression of genes responsible for the utilization of non-preferred nitrogen sources. AreB does not repress AreA-dependent genes directly but can act as a positive and negative regulator of nitrogen-regulated genes in *F. fujikuroi* [178]. Next to this, AreA and AreB functions are critical for wild type-like secondary metabolism. Both TFs directly bind and interact with the underlying DNA by a structural motif found in cluster promoter regions, such as *GA* or *BIK* [38,179]. GA was the first fungal SM shown to be strictly FfAreA-dependent [37,38]. Here, deletion of both *FfAREA* and *FfAREB* led to abolished GA biosynthesis and suggested synergistical activation [178]. Next, deletion of both resulted in the downregulation of six out of seven co-regulated genes of the *GA* gene cluster, which was accompanied by drastically reduced levels of H3K9ac at this cluster [38,39]. This suggests a dynamic interplay between *FfAREA* and *FfAREB* and HATs/HDACs [39]. Next to this, FUM biosynthesis in *F. fujikuroi* is regulated by an intrinsic cluster-specific TF (*FUM21*) and depends on the presence of both FfAreA and FfAreB. Here, constitutive overexpression of *FUM21* in the ∆∆*ffareA*/*ffareB* double mutant showed partial restoration of FUM biosynthesis, indicating positive regulation by nitrogen availability [180].

The velvet protein family is a fungal-specific conserved complex important for the regulation of secondary metabolism in filamentous fungi [25]. The complex consists of several subunits, i.e., VeA/Vel1/Ve1, VelB/Vel2 and VelC, in different *Fusarium* species. Furthermore, interaction between VeA/Vel1 and the putative methyltransferase Lae1 were determined [26,28,32,181,182]. In *F. oxysporum*, the combinatorial roles of FoAreA and velvet complex proteins was investigated. Down-regulation for BEA biosynthesis was observed in *FoAREA*, *FoVEA* and *FoLAE1* deletion strains; however, interaction studies could not determine physical interactions between FoAreA and velvet complex components [27]. For *F. oxysporum,* modification of chromatin accessibility in different SMBGCs was tested. Here, for triacetylfusarinine C and ferricrocin chromatin, accessibility in the ∆*fovelA* and ∆*fovelB* was completely abolished, while for ∆*folae1,* a strong reduction in accessibility was observed, similarly to what was seen for BIK biosynthesis [28]. Deletion of *FvLAE1* led to a dramatic change in global gene expression, where about 55% of all genes were de-regulated; most were classified as SM-regulatory [35]. Notably, *F. fujikuroi* transcriptomic data suggested different SM-regulation mechanisms in the ∆*ffvel1* deletion strain [26,55,73]. Downregulation of the *FUM*, *FUS*, *FUB, GPY* and *GA* genes and upregulation of *BIK* biosynthetic genes was observed [26,55,73] Similarly, deletion of *FfVEL2* caused reduced *GA*, *GPY* and increased *BIK* gene expression [26,55].

For *F. fujikuroi*, BIK biosynthesis depends on the presence of the global regulators FfPacC and FfAreA. PacC is a Cys_2_His_2_-zinc finger transcription factor involved in the pH-dependent expression of SM genes [183]. PacC is activated under basal to alkaline pH conditions, activating alkaline-expressed genes, while AreA controls nitrogen availability [177,183]. FfPacC represses BIK biosynthesis in basal medium and, to a certain extent, in acidic conditions, while FfAreA influences BIK biosynthesis indirectly, despite the presence of AreA DNA-binding motifs in the *BIK* promoters [179]. Interestingly, for *F. fujikuroi*, *FfVEL1* deletion overruled FfPacC/FfAreA dependency, since BIK is accumulated under unfavorable pH conditions and sufficient nitrogen conditions, despite unchanged transcript levels of FfPacC and FfAreA/FfAreB in the deletion strains [26]. However, the PacC-mediated repression of BIK was still maintained in an alkaline milieu in the *FfHDA1* deletion mutant, while the impact of the nitrogen-responsive TF FfAreA was abolished for inducing conditions [175]. Studies on targeted SMBGCs, such as the *BIK* gene cluster, demonstrate the versatile regulatory network underlying SM gene expression. As Vel1/Lae1 was shown to be involved in the modulation of chromatin accessibility in *F. oxysporum*, it is evident that similar mechanisms take place in *F. fujikuroi* [28]. In the case of BIK, it seems that FfVel1 acts upstream of FfAreA/FfPacC, putatively interacting directly with the chromatin structure or recruiting external factors and leading to the expression of the pigment in unfavorable conditions.

An extensive characterization of FfLae1, in *F. fujikuroi*, showed that 22 out of 48 SMBGCs were affected by transcriptomic analysis. Intriguingly, overexpression led to the activation of clusters of thus far unknown products, i.e., STC7 and PKS9 [35,55,73], as well as the otherwise silent *BEA* gene cluster [34,70]. For BIK and FSR, overexpression or deletion of *FfLAE1* led to the circumvention of other regulatory circuits, e.g., nitrogen or pH repression, which was shown before for BIK biosynthesis in the ∆*ffvel1* strain [26,34]. However, the overexpression of *FfHAT1* was able to partially restore the chemical profile of ∆*fflae1* [34].

Sge1 is considered a transcriptional regulator in higher eukaryotes, revealing important functions as a morphological switch between saprophytic and pathogenic lifestyle as well as for the regulation of fungal secondary metabolism [184,185]. Transcriptomic data of *F. fujikuroi* ∆*ffsge1* showed the de-regulation of 12 histone-modifying enzymes in nitrogen-sufficient conditions, which are preferred for *FfSGE1* expression [185]. This suggests a role of Sge1 in the transcriptional regulation of chromatin-modifying enzyme-encoding genes, which putatively leads to chromatin alterations. As shown previously, ∆*ffvel1* and ∆*ffsge1* led to increased and decreased BIK production, respectively. However, constitutive overexpression of *FfSGE1* in ∆*ffvel1* could not rescue BIK biosynthesis. Next, a *ffareA*-deficient strain showed loss of GA and FUM biosynthesis; however, overexpression of *FgSGE1* in this background could partially restore GA biosynthesis, while this was not the case for FUM. Similarly, overexpression of *FfSGE1* in the ∆*ffapf2* background (apicidin F (APF) cluster-specific TF), which abolishes APF biosynthesis, was able to partially restore APF biosynthesis [185].

To sum up, the dynamic interaction between regulators, TFs and/or chromatin-modifying enzymes appears manifold. Currently, the complex hierarchy of SM gene expression is still opaque, giving rise to a platform for future work in order to further unravel the exact mode of action of the different regulatory components and how they are interconnected to ensure the timely and reversible expression of SM genes in the genus *Fusarium.*

## 5. Conclusions

This review summarizes the latest advances in understanding chromatin regulatory mechanisms and underlines the importance of the chromatin structure on SM biosynthesis in the genus *Fusarium*. Furthermore, the versatile interplay in changes of the chromatin structure and the recruitment of transcription factors, or “regulators”, will help to deepen our understanding of the complex regulatory networks behind SM gene expression. Some histone marks seem to be more important than others for fungal secondary metabolism, e.g., Kmt6 (H3K27me3) or GCN5 (H3K4, H3K9, H3K18, H3K27 and H3K36ac), both considered important mediators for the expression of secondary metabolism-associated genes. However, even with histone methylation and acetylation, which appear to be already well-characterized in the genus *Fusarium*, their molecular function is not well-understood. Genome-wide distribution studies, combined with detailed molecular and chemical characterizations, are due to shed further light on their role and their functional network in detail. Additionally, there is still a series of predicted proteins with unknown function, as well as other possible histone marks such as phosphorylation, ubiquitinoylation or crotonylation, which are so far not examined at all. Furthermore, global regulators such as Lae1 and Sge1 were shown to either modify DNA accessibility directly or by interacting with other histone-modifying enzymes. However, little is known so far about the crosstalk between histone marks and “regulators”, providing an incentive for future research. Knowledge about the molecular aspects that govern SM gene regulation thus constitutes a powerful tool to gain broader insights into the deduction of novel compounds and to provide tools for novel cropping strategies.

## Figures and Tables

**Table 1:** Overview of predicted histone-modifying enzymes involved in histone methylation and acetylation as determined by InterPro [48].

	InterPro Accession Number	*F. fujikuroi*	*F. oxysporum*	*F. graminearum*	*F. solani*
***Histone lysine methyltransferases***					
**SET-domain**	IPR001214	25	37	26	23
**DOT-domain**		1	1	1	1
***Histone lysine demethylases***					
**JmjC-domain**	IPR003347	10	28	16	10
***Histone reader proteins***					
**Chromo/** **Chromoshadow-** **domain**	IPR000953	11	49	13	8
**BAH-domain**	IPR001025	4	15	8	4
***Histone acetyltransferases***					
**GNAT-domain**	IPR000182	63	99	63	72
**MYST-domain**	IPR040706	3	6	4	3
Other *	IPR022591 IPR013178	2	8	3	2
***Histone deacetylases***					
**Zn^2+^-dependent**	IPR023801	4	7	6	4
**Sirtuins**	IPR026590	8	15	9	9

* Sum of TAF1 and RTT109 domain-containing proteins.

**Table 2:** The table depicts known *Fusarium* metabolites and chromatin regulators (bold letters) which are shown to influence their biosynthesis in axenic culture. n.p. denotes that the key enzyme-encoding gene is not present in the *Fusarium* species; ? indicates an unknown regulatory mechanism behind the respective SM gene expression.

Gene Name	Product	*Fusarium* spp.				Literature
		*F. fujikuroi*	*F. graminearum*	*F. verticillioides*	*F. mangiferae*	
**NRPSs**						
**NRPS 1**	Malonichrom	n.p.	Kmt6	?	?	[61]
**NRPS 2**	Ferricrocin	Gcn5, Hda1	Kmt6	?	?	[53,61]
**NRPS 4**	Fusahexin	Kmt2, Kmt3, Ash1, Kmt6 Kdm5, Gcn5	Kmt6	?	?	[52]
**NRPS 5**	Fusaoctaxin	n.p.	Kmt6	n.p.	n.p.	[62]
**NRPS 6**	Fusarinine	Kmt2, Kmt3	Kmt6, Gcn5, Sas3, RID/Dim-2	?	?	[54]
**NRPS7**	Fusaristatins	n.p.	Kmt6, RID/Dim-2	n.p.	n.p.	[63,64]
**NRPS 8**	Gramilin	n.p.	Kmt6, Gcn5 RID/Dim-2	n.p.	n.p.	[65]
**NRPS 9**	Fusaoctaxin	n.p.	Kmt6	n.p.	n.p.	[62]
**NRPS 14**	Chrysogine	n.p.	Kmt6, Kdm5	n.p.	n.p.	[66]
**NRPS 17**	Ferrichrome	Gcn5	?	?	n.p.	[54]
**NRPS 22**	Beauvericin	Ash1, Kmt6, Gcn5, Hda1	n.p.	?	Kmt1	[67,68,69]
**NRPS 31**	Apicidin F	Kmt2, Kmt3, Ash1, Kmt6 Kdm5, Gcn5	n.p.	n.p.	n.p.	[70]
**NRPS 32**	Ferrirhodin	n.p.	n.p.	n.p.	n.p.	[71]
**NRPS 34**	Fusaric acid	Kmt2, Kmt3, Ash1, Kmt6, Hat1, Gcn5, Hda1, Hda2	n.p.	?	?	[72,73,74]
**PKSs**						
**PKS 1/NRPS**	Equisetin/ trichosetin	Kmt2, Kmt6, Gcn5	n.p.	?	?	[75]
**PKS 3**	Fusarubins	Kmt2, Kdm5, Ash1, Kmt6, Hda2	Kmt6, RID/Dim-2	?	?	[51]
**PKS 4**	Bikaverin	Kmt2, Kmt5, Kdm5, Ash1, Gcn5, Hda1, Hda2	n.p.	Kmt3,	?	[76]
**PKS 6**	Fusaric acid	Kmt2, Kmt3, Ash1, Kmt6, Hat1, Gcn5, Hda1, Hda2	n.p.	?	?	[72,73,74]
**PKS 10/NRPS**	Fusarin C	Kmt2, Kmt2, Kmt3, Kmt5, Kdm5, Ash1, Gcn5, Hda1, Hda2	Kmt5, Kmt6, Kdm5		?	[72]
**PKS 11**	Fumonisins	Kmt2, Kmt3, Ash1, Kmt6, Gcn5, Hda1	n.p.	Kmt1, Kmt2, Kmt3	n.p.	[72]
**PKS 13**	Gibepyrones	Kmt3, Ash1, Gcn5, Hda1	Kmt6	?	?	[55]
**PKS 19**	Fujikurins	Kmt6	n.p.	n.p.	n.p.	[77]
**PKS22**	Zearalenone	n.p.	Kmt5, Kmt6, Gcn5, Sas3	n.p.	n.p.	[78]
**PKS23**	Fusaristatins	n.p.	Kmt6	n.p.	n.p.	[63,64]
**PKS24**	Fusarielins	n.p.	Kmt6, Kdm5, Sas3	n.p.	n.p.	[79,80,81,82]
**PKS25**	Aurofusarin	n.p.	Kmt2, Kmt6, Gcn5, Sas3, Hep1, RID/Dim-2	n.p.	n.p.	[83]
**PKS26**	Zearalenone	n.p.	Kmt5, Kmt6, Gcn5, Sas3	n.p.	n.p.	[78]
**PKS27**	Orcinol	n.p.	Kmt6, GCN5, Sas3, RID/Dim-2	n.p.	n.p.	[84]
**PKS 39**	Depudecin	n.p.	n.p.	?	n.p.	[85]
**PKS 40**	Fusapyrone	n.p.	n.p.	?	Kmt1	[86,87]
**DMATSs**						
**DMATS1**	r-N-DMAT	Gcn5	n.p.	?	?	[88]
**TCs**						
**DTC1-1**	Gibberellins	Kmt2, Ash1, Kmt5, Kmt6, HAT1, Gcn5, Hda1, Hda2	n.p.	n.p.	?	[89]
**TeTC1**	Carotenoids	Kmt2, Kmt3, Ash1, Kmt6, Gcn5	Kmt6, Gcn5, Sas3, RID/Dim-2	?	?	[90]
**STC1**	Germacrene	Kmt2, Kmt3, Ash1, Kmt6, Kdm5, Gcn5, Hda1	?	?	?	[91]
**STC3**	Eremophilene	Ash1	n.p.	?	?	[91]
**STC4**	Koraiol	Kmt2, Ash1, Gcn5	Kmt6	?	?	[92]
**STC5**	Guaiadiene	Kmt6, GCN5	Kmt6	n.p.	?	[91]
**STC6**	Acorenol	Kmt6	Kmt6	?	?	[92]
**STC9**	Culmorin	Gcn5	Kmt6	?	?	[93]
**TRI5**	Deoxynivalenol	n.p.	Kmt2, Kmt5, Kmt6, Kdm5, Gcn5, Sas3, Elp3, Hep1, Hda2, RID/Dim-2	n.p.	n.p.	[23]
**FUP1**	Fusaproliferin	n.p.	n.p.	n.p.	?	[60]
